# Abundance and Niche Differentiation of Comammox in the Sludges of Wastewater Treatment Plants That Use the Anaerobic–Anoxic–Aerobic Process

**DOI:** 10.3390/life12070954

**Published:** 2022-06-24

**Authors:** Sheng-Nan Zhang, Jian-Gong Wang, Dan-Qi Wang, Qiu-Yue Jiang, Zhe-Xue Quan

**Affiliations:** Ministry of Education Key Laboratory for Biodiversity Science and Ecological Engineering, National Observations and Research Station for Wetland Ecosystems of the Yangtze Estuary, Institute of Biodiversity Science and Institute of Eco-Chongming, School of Life Sciences, Fudan University, Shanghai 200433, China; 19210700131@fudan.edu.cn (S.-N.Z.); 14110700066@fudan.edu.cn (J.-G.W.); 18110700020@fudan.edu.cn (D.-Q.W.)

**Keywords:** nitrification, comammox, wastewater treatment plants, dissolved oxygen, bacterial community

## Abstract

Complete ammonia oxidizers (comammox), which directly oxidize ammonia to nitrate, were recently identified and found to be ubiquitous in artificial systems. Research on the abundance and niche differentiation of comammox in the sludges of wastewater treatment plants (WWTPs) would be useful for improving the nitrogen removal efficiency of WWTPs. Here, we investigated the relative abundance and diversity of comammox in fifteen sludges of five WWTPs that use the anaerobic–anoxic–aerobic process in Jinan, China, via quantitative polymerase chain reaction and high-throughput sequencing of the 16S rRNA gene and ammonia monooxygenase gene. In the activated sludges in the WWTPs, comammox clade A.1 was widely distributed and mostly comprised *Candidatus* Nitrospira nitrosa-like comammox (>98% of all comammox). The proportion of this clade was negatively correlated (*p* < 0.01) with the dissolved oxygen (DO) level (1.7–8 mg/L), and slight pH changes (7.20–7.70) affected the structure of the comammox populations. *Nitrospira* lineage I frequently coexisted with *Nitrosomonas*, which generally had a significant positive correlation (*p* < 0.05) with the DO level. Our study provided an insight into the structure of comammox and other nitrifier populations in WWTPs that use the anaerobic–anoxic–aerobic process, broadening the knowledge about the effects of DO on comammox and other nitrifiers.

## 1. Introduction

Nitrification, a crucial process linking nitrogen fixation and denitrification, plays a crucial role in the nitrogen cycle [1]. It consists of two sequential steps. First, ammonia is converted to nitrite by ammonia-oxidizing bacteria (AOB) [2] or ammonia-oxidizing archaea (AOA) [3]. Next, nitrite is oxidized into nitrate by nitrite-oxidizing bacteria (NOB) [4,5].

Complete ammonia oxidizers (comammox) [6] have been recently identified, overturning the long-term canonical conception of nitrification [7,8,9]. Comammox can directly oxidize ammonia to nitrate via nitrite and have a high growth yield with a low growth rate [6,10]. Currently known comammox are affiliated with *Nitrospira* lineage II, previously known as strict-NOB (sNOB), including *Nitrospira inopinata* [7], *Candidatus* Nitrospira nitrosa [8], *Candidatus* Nitrospira nitrificans [8], and *Candidatus* Nitrospira kreftii [9]. Comammox can be split into clade A and clade B [7,11]; clade A can be further classified into clade A.1 and clade A.2 based on the amino acid sequence of ammonia monooxygenase subunit A (AmoA) [12]. Comammox have been ubiquitously found in natural and artificial ecosystems, such as salt marshes [13], drinking water systems [14], grasslands [15], and lake sediments and forest soils [12] based on *amoA* gene amplicon and metagenome sequencing.

Our current lifestyle causes an excessive discharge of nutrients, including nitrogenous compounds, into the water ecosystems, eventually leading to eutrophication. To prevent eutrophication, which can cause oxygen depletion and disturb the ecological balance [16], decreasing the level of nutrient salts in sewage streams is a pressing need. The anaerobic–anoxic–aerobic (A^2^O) process has been broadly applied in wastewater treatment plants (WWTPs) to efficiently remove phosphorus and nitrogen from inflowing wastewater [17,18]. Nitrification is an essential process at the aeration stage of WWTPs, where canonical nitrifying microorganisms are the dominant autotrophic functional microbes [19]. The characteristics and contribution of canonical nitrifiers in WWTPs have been broadly studied [20,21,22] as well as the effects of several environmental factors on canonical nitrifiers. For example, the ammonium concentration, dissolved oxygen (DO) concentration, and temperature have specific selectivity for AOB and AOA in WWTPs [23,24]; distinct AOB are dominant ammonia oxidizers in WWTPs with a high salinity [25], and heavy metals in WWTPs have a more significant negative effect on AOB than on sNOB [26].

Recently, researchers have focused on the characteristics of comammox in WWTPs. Based on reverse transcription-quantitative polymerase chain reaction (PCR) results, comammox have been found to greatly contribute to ammonia oxidation in WWTPs [27]. Some studies have determined that comammox prefer environments with a low ammonium concentration because of their relatively high affinity for ammonia [28,29]. Additionally, comammox are dominant ammonia oxidizers in WWTPs with relatively long solid retention times because of their slow growth rate [28,29,30]. Wastewater resources affect the distribution of comammox, which are more abundant in municipal wastewater than in refinery wastewater [31]. Furthermore, some researchers found that comammox are the dominant nitrifiers under limited DO concentrations (<1 mg/L), whereas traditional nitrification in WWTPs requires relatively high DO concentrations (>2 mg/L) [30]. The nitrification at low DO levels could considerably reduce energy costs in WWTPs and improve their efficiency because denitrification could occur simultaneously under relatively low DO concentrations (<0.75 mg/L) [32,33].

Compared with studies on canonical nitrifiers, studies on the distribution and composition of comammox in WWTPs are scarce. Moreover, the impacts of environmental factors on comammox and the relationships between comammox and other nitrifiers in WWTPs require further exploration [11,27,34]. In this study, we hypothesized that the abundance and niche differentiation of comammox in the sludges of WWTPs that use the A^2^O process might be affected by DO levels. Therefore, we collected fifteen sludges from five WWTPs in Jinan, China, and analyzed their physicochemical parameters. This study aimed to reveal the abundance and distribution of comammox and other nitrifiers, as well as the effects of possible environmental factors on them, while confirming the comammox presence and community structure in WWTPs that use the A^2^O process.

## 2. Materials and Methods

### 2.1. Sample Collection and Physicochemical Analysis

Sludge samples were collected from the anoxic stage (A2), inlet (O1), and outlet (O2) of the aeration stage in five WWTPs (A, B, C, D, and E) in Jinan, Shandong Province, China, in October 2017. The collected samples were stored at 4 °C.

Temperature, DO, and pH were measured using a YSI ProODO Portable Dissolved Oxygen instrument (YSI, Yellow Springs, OH, USA). Sample supernatants were filtered through 0.22 μm filters (Merck Millipore, Billerica, MA, USA). The concentration of ammonium was measured using the modified indophenol method [35,36], and the concentrations of nitrite and nitrate were measured using a Dionex ICS-1100 ion chromatograph (Thermo Fisher Scientific, Waltham, MA, USA).

### 2.2. DNA Extraction and Quantitative PCR (qPCR)

Genomic DNA was extracted from 0.25 g of wet sludge using a Dneasy PowerSoil Kit (Qiagen, Hilden, Germany). After assessment of the quality by agarose gel electrophoresis, the concentration of genomic DNA was determined using a Qubit 2.0 (Thermo Fisher Scientific).

qPCR was performed to assess the abundance of total bacteria, AOB, sNOB, comammox clade A, comammox clade B, and AOA using the FastStart Universal SYBR green master mix (Rox) (Roche, Mannheim, Germany). The reaction volumes and procedures of partial nested PCR were performed as previously described [12]. Genomic DNA was diluted 10-fold to avoid interference from environmental factors [37]. qPCR primers used in this study are listed in Table S1. The results were analyzed using MxPro qPCR software (version 3.0) (Agilent Technologies, Santa Clara, CA, USA), according to the manufacturer’s instructions. The construction of standard plasmids and standard curves was performed as previously reported [12,37].

### 2.3. High-Throughput Sequencing of the 16S rRNA Gene and Comammox amoA Gene

The V1–V2 regions of the bacterial 16S rRNA gene were amplified to investigate the bacterial community following these PCR procedures: 94 °C for 5 min; 35 cycles of 95 °C for 30 s, 55 °C for 50 s, and 72 °C for 50 s; and then 72 °C for 5 min. The reaction volume consisted of 18 μL of sterilized water, 2 μL of primers (10 μM), 2 μL of DNA (3–5 ng), 1 μL of bovine serum albumin (BSA, 20 mg/μL), and 25 μL of Ex Taq premix (Takara Bio, Kusatsu, Shiga, Japan). The primers used are listed in Table S2. Partial nested PCR was conducted to amplify the comammox *amoA* gene. In the first step of amplification, primers A189Y/C576R were used. The obtained PCR products were then used as template DNA and were amplified using the primer pair CA209F/C576R-barcodes [12]. The reaction volumes and procedures of partial nested PCR were previously described [12]. The specific information of the primers used for high-throughput sequencing is provided in Table S2. Barcodes (12 bp) were linked to the 5’-end of the primers to identify the sequence sources. After PCR products with the specific length were retrieved using the AxyPrep DNA Gel Extraction Kit (Axygen, Tewksbury, MA, USA), same-gene products were individually mixed at equal concentrations for library construction using the KAPA LTP Library Kit (KAPA Biosystems, Boston, MA, USA). Next-generation sequencing (NGS) technology was performed using an Illumina HiSeq platform (PE250; Illumina, San Diego, CA, USA).

### 2.4. Data Processing and Statistical Analyses

Sequencing data processing was performed according to a previous report [12]. Briefly, the quality of the raw data was controlled using FastQC (http://www.bioinformatics.babraham.ac.uk/projects/fastqc/, accessed on 12 April 2021); qualified sequences were then processed using QIIME software (version 1.8) (http://www.qiime/org, accessed on 12 April 2021) [38]. After removal of the 12-bp barcodes, sequences were distinguished by their barcodes and assigned to individual samples. USEARCH61 software [39] was used to check chimeras of the 16S rRNA gene and comammox *amoA* gene sequencing data against the SILVA 16S rRNA gene database [40,41] and improved related copper-containing membrane monooxygenase (CuMMO)-associated gene database [42], respectively. Operational taxonomic units (OTUs) of the 16S rRNA gene and comammox *amoA* gene (comammox OTUs) were clustered based on a 97% and a 90% similarity cutoff, respectively, against the corresponding database. Phylogenetic analyses based on maximum likelihood were then conducted to further obtain the classified information of these OTUs.

Most statistical analyses were conducted in R (version 4.0.3, R Foundation for Statistical Computing, Vienna, Austria). The “ggpubr” package was used to perform the variation analysis, and the Kruskal–Wallis test was performed to analyze the differences in the physicochemical parameters and nitrifier abundance among groups. The “dunn.test” package was used to determine specific differences between groups. The “Hmisc” package was used to calculate the Spearman correlations between the environmental factors, and the different OTUs were divided into *Nitrospira* (*Nitrospira* OTUs) and *Nitrosomonas* (*Nitrosomonas* OTUs). The network used to visualize Spearman’s correlations was built in Gephi software (version 0.9). GraphPad Prism (version 9, GraphPad Software Inc., La Jolla, CA, USA) was used to investigate the linear relationships between environmental factors and various nitrifiers in the WWTPs. The maximum-likelihood phylogenetic trees were constructed using MEGAX [43,44] and were visualized using iTOL [45]. Statistical significance was considered at *p* < 0.05.

### 2.5. Sequence Accession Numbers

The raw high-throughput sequencing data were deposited in the National Omics Data Encyclopedia (NODE) and NCBI GenBank Sequence Read Archive (SRA) under project ID OEP003205 (https://www.biosino.org/node/project/detail/OEP003205, accessed on 13 and 14 March 2022) and accession number PRJNA816657 (https://www.ncbi.nlm.nih.gov/sra/PRJNA816657, accessed on 19 March 2022), respectively.

## 3. Results

### 3.1. Physicochemical Parameters of the WWTPs

The temperature, DO, and pH of the five WWTPs were measured in the field; the other parameters were measured in the laboratory (Table 1). Differences in these parameters among the different stages are shown in Figure S1. Generally, except for nitrate and temperature, the physicochemical parameters at the anoxic stage were significantly different from those at the aeration stage (*p* < 0.05, Kruskal–Wallis). However, the parameters were similar at the inlet and outlet of the aeration stage. The pH at the anoxic stage (6.83 ± 0.07) was lower than that at the inlet (7.41 ± 0.19) and outlet (7.43 ± 0.18) of the aeration stage. The DO concentration at the anoxic stage (0.38 ± 0.13 mg/L) was significantly lower than that at the inlet (6.00 ± 2.19 mg/L) and outlet (6.94 ± 3.95 mg/L) of the aeration stage. At the aeration stage, D-O1 had the highest DO level (8.00 mg/L), while B-O2 had the lowest level (1.70 mg/L). At the anoxic stage, the highest DO levels were found in D-A2 and E-A2 (0.5 mg/L). The concentrations of nitrite in these samples were not detectable.

### 3.2. Abundance of Comammox and Other Nitrifiers in WWTPs

**Figure 1:**
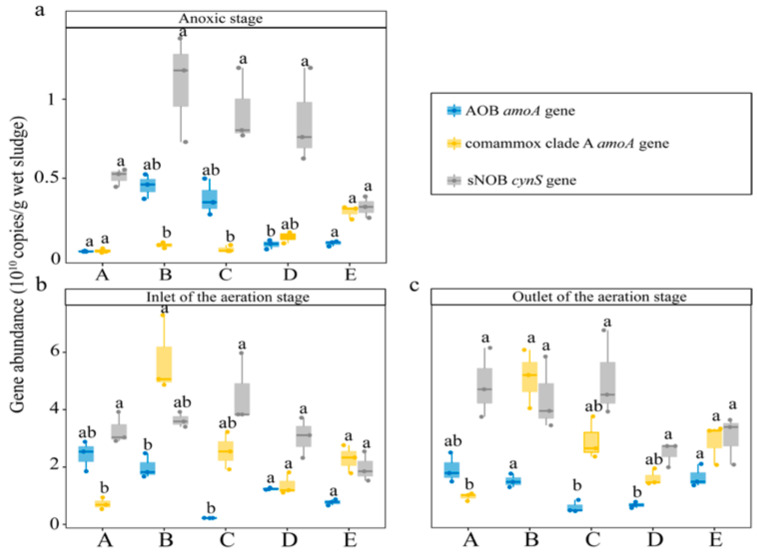
Abundance of comammox and other nitrifiers in samples collected from the anoxic stage (**a**), inlet of the aeration stage (**b**), and outlet of the aeration stage (**c**) of plants A, B, C, D, and E. Dunn’s test was used to verify the variation among the specific groups if the Kruskal–Wallis test showed significant differences. The letters “a” and “b” indicate significant differences (*p* < 0.05). Error bars indicate the standard error of the mean of three replicates.

qPCR revealed that comammox clade A, AOB, and sNOB were ubiquitous in the analyzed sludges (Figure 1). AOA and comammox clade B were not detected in the samples, which might have resulted from the abundance of these nitrifiers being below the detection limit. Based on the Kruskal–Wallis test, the proportion of comammox clade A varied significantly among samples (*p* < 0.05) (Figure 1). At the anoxic stage (Figure 1a), sNOB were the dominant nitrifiers, and comammox clade A had a relatively low proportion (0.10 to 0.80%). However, comammox clade A had a relatively high proportion compared with all AOB, except B-A2 and C-A2.

At the aeration stage (Figure 1b,c), comammox clade A generally had a higher proportion than AOB, except in plant A. sNOB also had a relatively high proportion in most samples. Moreover, the percentage of comammox clade A was similar to that of sNOB in some plants. In plant B, the abundance of comammox clade A (5.42 ± 1.12 × 10^10^ copies/g wet sludge) was slightly higher than that of sNOB (4.02 ± 0.92 × 10^10^ copies/g wet sludge). Meanwhile, the abundance of comammox clade A (2.59 ± 0.63 × 10^10^ copies/g wet sludge) was similar to that of sNOB (2.50 ± 0.85 × 10^10^/g wet sludge) in plant E.

### 3.3. Composition and Distribution of Nitrospira and Nitrosomonas in WWTPs

The proportions of nitrifiers in the total bacterial community were investigated based on the 16S rRNA gene (Figure S2a). *Nitrospira* and *Nitrosomonas* presented relatively high proportions in all samples collected from the aeration stage, representing 9.69% and 1.57% on average, respectively. Except for those two genera, other nitrifiers (such as *Nitrotoga* and *Nitrosococcus*) only appeared in specific samples at low proportions. Therefore, the ubiquitous *Nitrospira* and *Nitrosomonas* were selected as representative nitrifiers in these samples.

The differences among the six *Nitrospira* lineages were clear, and all *Nitrospira* OTUs belonged to *Nitrospira* lineage I or II based on the phylogenetic analysis. Currently, all members of *Nitrospira* lineage I are all classified as sNOB. However, both comammox and partial sNOB can be classified as *Nitrospira* lineage II and are hardly distinguished by their 16S rRNA gene [7,8,9]. The retrieved sequences belonging to *Nitrospira* lineage I and II accounted for 86.13% and 13.87%, respectively, of all *Nitrospira* sequences. Therefore, the majority of sNOB in WWTPs could be classified as *Nitrospira* lineage I.

**Figure 2:**
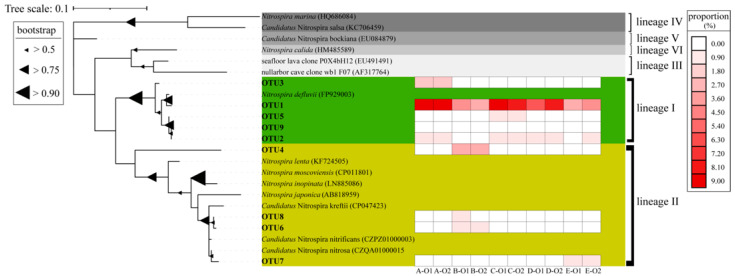
Composition and distribution of major *Nitrospira* OTUs in samples from the aeration stage of the WWTPs. The phylogenetic tree was constructed based on all 16S rRNA gene sequences classified as *Nitrospira* OTUs and accounting for more than 0.10% of all reads on average. The colors in the rectangular grids indicate the proportion of *Nitrospira* OTUs relative to total bacteria.

Furthermore, the major *Nitrospira* OTUs (accounting for more than 0.10% of all bacteria on average) were chosen for further phylogenetic analysis to reveal the composition of primary *Nitrospira* in these samples at the aeration stage of the WWTPs. As shown in Figure 2, *Nitrospira* OTU1, belonging to *Nitrospira* lineage I, was the dominant *Nitrospira* in most samples, accounting for 6.28% of all bacteria (65.42% of all *Nitrospira* sequences). *Nitrospira* OTU4 and *Nitrospira* OTU6 were the major OTUs of *Nitrospira* lineage II, accounting for 36.42% and 24.49%, respectively.

The ratios of *Nitrospira* lineages and *Nitrosomonas* to all nitrifiers (including *Nitrospira* and *Nitrosomonas*) are shown in Figure S2b. *Nitrospira* lineage I was the dominant nitrifier in all plants, with the exception of plant B. The highest ratio of *Nitrospira* lineage I to total nitrifiers (89.45%) was obtained in A-O2, where the ratios of *Nitrospira* lineage II and *Nitrosomonas* to all nitrifiers accounted for 1.72% and 8.83%, respectively. The highest ratio of *Nitrospira* lineage II to all nitrifiers (45.86%) was obtained in B-O1, whereas the ratios of *Nitrospira* lineage I and *Nitrosomonas* accounted for 43.67% and 10.47%, respectively. *Nitrosomonas* was not the dominant nitrifier in any sample, and its highest ratio to all nitrifiers (20.61%) was obtained in D-O1, corresponding to one-quarter of that of *Nitrospira*.

### 3.4. Correlation of Comammox and Other Nitrifiers with Environmental Parameters

**Figure 3:**
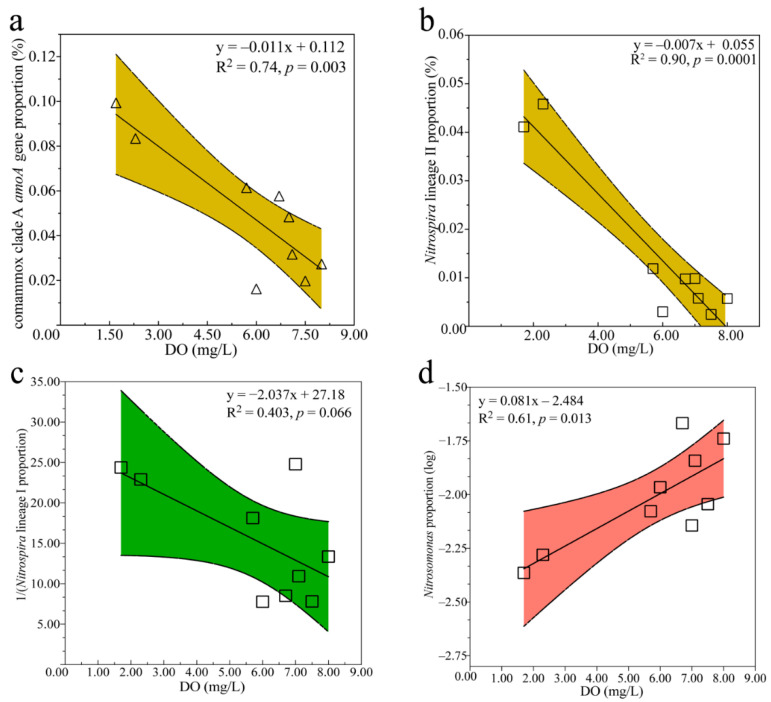
Correlations between the DO levels and proportions of comammox clade A (**a**), *Nitrospira* lineage II (**b**), *Nitrospira* lineage I (**c**), and *Nitrosomonas* (**d**) in all bacteria. Colors in the graphs show the 95% confidence interval. Data in (**a**) (shown by triangles) were obtained from the qPCR results, while data in other graphs (showed by squares) were obtained from the 16S rRNA gene sequencing data. The outlier shown in Table 1 was eliminated.

The proportion of comammox clade A relative to total bacteria varied significantly among the different samples collected from the aeration stage of the WWTPs (Figure 1b,c). Linear correlation analysis performed on the qPCR data indicated that comammox clade A presented high proportions of all bacteria under low DO levels (R^2^ = 0.53, *p* < 0.05) (Figure 3a). Spearman analysis conducted on the 16S rRNA gene sequencing results further indicated that the DO levels had specific effects on the composition of several nitrifiers (Figure S3). The relative abundance of *Nitrospira* lineage II declined significantly with increasing DO levels (R^2^ = 0.90, *p* < 0.01) (Figure 3b), whereas the relative abundance of *Nitrospira* lineage I tended to increase with increasing DO levels (R^2^ = 0.403) (Figure 3c). According to this correlation, the relative abundance of *Nitrospira* had the tendency to increase with increasing DO levels (Figure S3). Moreover, the proportion of the majority of *Nitrosomonas* OTUs (accounting for 79.39% of all *Nitrosomonas*) significantly increased with increasing DO levels (R^2^ = 0.61, *p* < 0.05) (Figure 3d).

### 3.5. Network Analysis of Nitrospira and Nitrosomonas Communities

**Figure 4:**
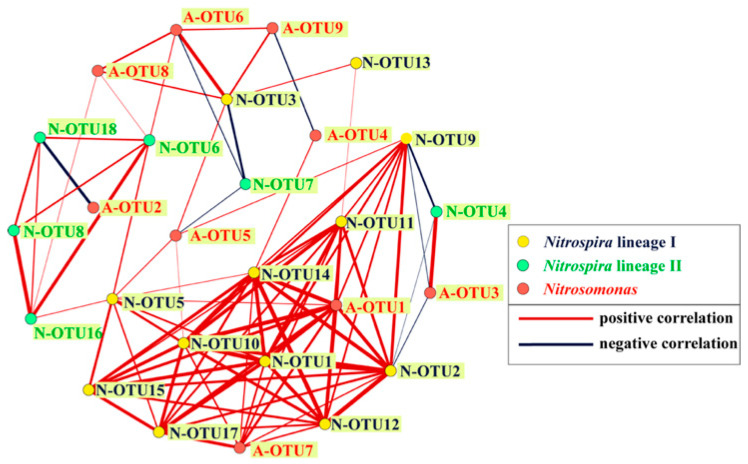
Network analysis of the correlations among the major nitrifier OTUs. These OTUs had at least 10 copies in each sample on average. “N OTU” and “A OTU” represent *Nitrospira* OTUs and *Nitrosomonas* OTUs, respectively. Significant correlations (*p* < 0.05, Spearman) were selected to present in the graph. The line thickness represents the weight of the correlations.

Network and Spearman correlation analyses were combined to analyze the specific and significant (*p* < 0.05) connections between *Nitrospira* lineages and *Nitrosomonas* with high abundance (Figure 4). *Nitrosomonas* OTU1, the dominant *Nitrosomonas* (accounting for 58.32% of all *Nitrosomonas*) showed close and positive correlations with most OTUs belonging to *Nitrospira* lineage I. In addition to *Nitrosomonas* OTU1, the majority of *Nitrosomonas* with relatively high abundance tended to have significant positive correlations with OTUs classified as *Nitrospira* lineage I. The correlations between OTUs classified as *Nitrospira* lineage II and *Nitrosomonas* OTUs were mostly negative. These results suggest that niche differentiation may exist between *Nitrospira* lineage I and lineage II and *Nitrosomonas.*

### 3.6. Phylogenetic Analysis and Relationships of Comammox OTUs with Environmental Factors

**Figure 5:**
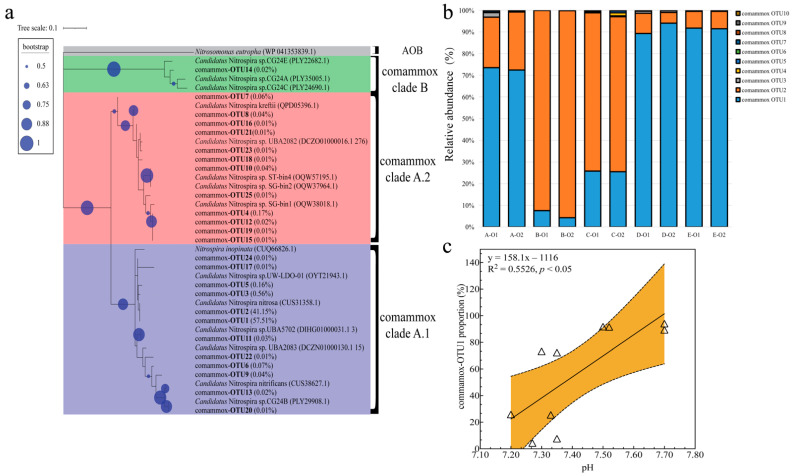
Comammox composition of the samples from the aeration stage of the WWTPs. (**a**) The maximum-likelihood phylogenetic tree was constructed by the retrieved AmoA amino acid sequence of comammox. The percentages in parentheses represent the percentages of comammox OTUs in all retrieved sequences. The 25 major OTUs (more than 0.01% of all reads) with a 90% nucleotide similarity cutoff are shown. (**b**) Distribution of the 10 major comammox OTUs at the aeration stage of the WWTPs. (**c**) Linear correlation between the ratio of comammox OTU1 to all comammox and pH. The triangles in (**c**) represent the data obtained from the partial nested PCR results. Abbreviations are consistent with those in Table 1.

A total of 270,617 sequences of the comammox *amoA* gene were retrieved via partial nested PCR (Table S3). The phylogenetic tree showed that comammox clade A.1 represented the dominant comammox in all samples from the aeration stage of the WWTPs, rather than comammox clade A.2 or comammox clade B (Figure 5a). Comammox OTU1 (reaching an average of 57.51%) and comammox OTU2 (reaching an average of 41.15%) were the primary OTUs in these samples from the aeration stage, and they were the closest to *Candidatus* Nitrospira nitrosa [8]. Comammox OTU1, comammox OTU2, and *Candidatus* Nitrospira nitrosa shared identical AmoA protein sequences. However, based on their nucleotide sequences, comammox OTU1 showed 90.05% and 89.55% similarity with comammox OTU2 and *Candidatus* Nitrospira nitrosa, respectively. Comammox OTU2 showed 96.52% similarity with *Candidatus* Nitrospira nitrosa.

The distribution of specific comammox in the samples from the aeration stage is shown in Figure 5b. At the aeration stages of plants B and C, comammox OTU2 was the dominant comammox, whereas comammox OTU1 was the dominant one in the other plants. Linear fitting analysis (Figure 5c) revealed that the ratio of comammox OTU1 to all comammox increased significantly with increasing pH (R^2^ = 0.55, *p* < 0.05). The analysis results suggested that a slight change in pH could affect the specific comammox composition of these samples.

## 4. Discussion

This study investigated the abundance of comammox and other nitrifiers in fifteen sludge samples collected from WWTPs that use the A^2^O process. After determining the abundance and distribution of the nitrifiers, we identified the dominant type of comammox in these samples. We also determined the correlations between important environmental factors and the compositions of comammox and other nitrifiers in samples from the aeration stage of the WWTPs.

The qPCR results revealed that AOB, comammox clade A, and sNOB coexisted in these sludge samples while AOA and comammox clade B were not detected (Figure 1). Comammox usually coexist with AOB and AOA [27,46] (Table 2), although AOA usually have a low abundance and only dominate in some WWTPs [46]. Comammox clade B is usually absent or present at low proportions in activated sludge [11,12].

The analyses combining the investigation of the conserved functional genes and the 16S rRNA gene revealed NOB as the dominant nitrifiers, and *Nitrospira* was the dominant genus classified as sNOB in the aeration stage of the WWTPs (Figure S2). Furthermore, *Nitrospira* OTUs could be divided into *Nitrospira* lineage I and lineage II (Figure 2), consistent with previous reports [47,48,49,50,51,52,53,54]. Other genera belonging to sNOB were present in specific samples at relatively low proportions. *Nitrobacter* is not a dominant genus in WWTPs, preferring a nitrogen-rich environment [54]. *Nitrotoga* may coexist with *Nitrospira* in WWTPs, but generally grow at low operating temperatures (from 10 to 17 °C) [55]. Compared with the results based on the 16S rRNA gene (Figure S2b), the proportions of AOB obtained from the qPCR results (Figure 1) were underestimated, perhaps due to the mismatch of the universal primers for AOB used in qPCR [56,57].

In a previous study, comammox clade A.1 and comammox clade A.2 were positively correlated with nitrate and total nitrogen concentrations, respectively [60], suggesting that different clades contribute differently to ammonia oxidation activity and should therefore be further researched regarding their structure in WWTPs. Comammox clade A.1, especially closely related with *Candidatus* Nitrospira nitrosa, dominated the comammox community in the samples from the aeration stage of the WWTPs (Figure 5). *Candidatus* Nitrospira nitrosa-like comammox, rather than other known comammox, are usually present at high proportions in the comammox populations of WWTPs [30,58], while *Candidatus* Nitrospira nitrificans-like comammox usually dominate drinking water treatment plants [47]. Transcriptome analyses of the comammox of WWTPs (Table 2) have suggested that *Candidatus* Nitrospira nitrosa-like comammox may greatly contribute to the ammonia-oxidizing activity at the aeration stage of WWTPs [27,46].

Our results revealed that the structure of comammox clade A.1 in the samples from the aeration stage varied (Figure 5b), and pH changes might be an important factor affecting the structure of comammox populations at the aeration stage of WWTPs (Figure 5c). These results, combined with previous reports on the impacts of pH on comammox in various ecosystems, suggest that pH might correlate with the structure of comammox populations [13,61,62].

In this study, the proportion of comammox clade A dominated by *Candidatus* Nitrospira nitrosa-like increased with decreasing DO levels (Figure 3a), corroborating previous reports [30,59,63]. *Candidatus* Nitrospira nitrosa were enriched in a bioreactor with a limited DO level [8]. Moreover, comammox genomes usually contain putative cytochrome *bd*-like oxidases [29], which have a high affinity for oxygen and can be expressed under oxygen-limited conditions [64,65,66]. Our results also indicated that comammox in WWTPs prefer environments with low DO levels. AOA would also not be restricted under low DO levels due to their high affinity for oxygen [67]. A distinct AOA in Nitrososphaeraceae was reported to greatly contribute to ammonia oxidation, surpassing the contribution of comammox in a WWTP with low DO levels and a high ammonium concentration [46].

The proportion of *Nitrospira* lineage I tended to increase with increasing DO levels, although the relationship was not statistically significant (Figure 3c). Research on *Nitrospira* lineage I has revealed that nitrate can be an alternative electron acceptor under deficient DO levels [68,69]. The potential ability of *Nitrospira* lineage I to survive under low DO levels based on genome analysis has also been reported [70]. In this study, the proportion of most *Nitrosomonas* increased with increasing DO levels (Figure 3d), which is consistent with a previous report [29]. Most AOB depend on the *aa3*-type heme–copper oxidase, which has a low affinity for oxygen [71], suggesting that DO might be a restrictive environmental factor for AOB. The characteristics of nitrifiers under low DO levels are not completely understood and thus need to be further explored.

The network constructed for the correlations among nitrifiers (Figure 4) revealed that the proportion of OTUs classified as *Nitrospira* lineage I generally had positive correlations with *Nitrosomonas* OTUs. *Nitrospira* lineage I usually coexist with AOB instead of *Nitrospira* lineage II, based on the different nitrite optima of the two lineages [49,52,53]. This suggests that the positive correlation between the *Nitrospira* lineage I proportion and DO levels might be affected by the positive correlation between *Nitrospira* lineage I and *Nitrosomonas* proportions. Overall, the discovery of comammox has added new and important information on the relationships of nitrifiers in WWTPs that require further in-depth research in the future.

## 5. Conclusions

In summary, *Candidatus* Nitrospira nitrosa-like comammox were ubiquitous and dominated (>98%) the comammox population in the aeration stage of the WWTPs. The DO levels (1.7–8.0 mg/L) were negatively correlated (*p* < 0.01) with the proportion of comammox clade A but positively correlated (*p* < 0.05) with the proportion of dominant *Nitrosomonas* in the activated sludges. *Nitrospira* lineage I, the dominant sNOB, generally coexisted with *Nitrosomonas* instead of *Nitrospira* lineage II. Slight pH variations (7.20–7.70) caused changes (*p* < 0.05) in the structure of the comammox populations at the aeration stage of the WWTPs. This study provided an insight into the structures of nitrifier populations in WWTPs and broadened the knowledge of the effects of DO levels on comammox and other nitrifiers. Further studies are needed to clarify the contribution of comammox, the structure of other nitrifiers, and the nitrifying efficiency of WWTPs under low DO levels.

## Figures and Tables

**Table 1:** Physicochemical parameters of samples collected from the WWTPs.

	Nitrate (mg/L)	Ammonium (mg/L)	Temperature (°C)	DO (mg/L)	pH
A-A2 ^1^	35.34	3.42	21.00	0.20	6.96
A-O1	68.20	1.44	20.80	6.00	7.30
A-O2	69.44	- ^2^	21.10	7.50	7.35
B-A2	89.90	14.04	19.90	0.40	6.82
B-O1	47.12	3.06	20.20	2.30	7.35
B-O2	83.70	-	19.90	1.70	7.27
C-A2	83.70	5.04	19.90	0.30	6.82
C-O1	106.02	1.80	19.70	6.70	7.20
C-O2	114.08	0.72	20.00	12.70 ^3^	7.33
D-A2	44.64	20.70	19.30	0.50	6.80
D-O1	73.78	8.10	19.20	8.00	7.70
D-O2	109.12	0.36	19.80	7.10	7.70
E-A2	88.66	4.50	19.30	0.50	6.77
E-O1	107.26	3.96	19.10	7.00	7.50
E-O2	80.60	0.18	19.10	5.70	7.52

^1^ A, B, C, D, and E are the five studied WWTPs in Jinan. A2 denotes the anoxic stage. O1 and O2 are the respective inlet and outlet of the aeration stage. ^2^ Undetectable value. ^3^ The value could be an outlier and therefore was eliminated in the following analyses.

**Table 2:** Previously reported compositions of different nitrifiers and their relationships with environmental factors in WWTPs.

Ammonium Inf ^1^	Ammonium Eff ^2^	DO	Temperature	pH	Finding	Reference
(mg/L)						
18.3–38.3	0.4–1.7	2–5.2	18.8–27.1	7.1–7.8	Comammox actively participate in ammonia oxidation in WWTPs.	[27]
118 ± 15	6 ± 2	0.07	21.2	6.7	AOA outnumber AOB or comammox and contribute the most to ammonia oxidation in WWTPs.	[46]
26.9–49.0	-	0.1–3.2	15–34	7.01–8.07	Comammox are the dominant ammonia oxidizers in WWTPs in different seasons.	[47]
-	0–10.3	0–0.4	-	7.0–7.5	Comammox are the dominant ammonia oxidizers in a sequencing batch reactor under low DO levels.	[58]
-	18.4 ± 4.9	0.2–1.0	20.3 ± 1.1	- ^3^	Comammox are the dominant ammonia oxidizers in nitrification systems at low DO levels.	[30]
-	0.69 ± 1.08	0.5	-	6.3–6.8	Comammox are the dominant ammonia oxidizers under weakly acidic and low DO levels.	[59]

^1^ Ammonium concentrations of influent wastewater. ^2^ Ammonium concentrations of effluent wastewater. ^3^ pH was not reported in this paper, but alkalinity was 4.6 ± 0.5 milliequivalent per liter.

## Data Availability

The raw high-throughput sequencing data are available from the National Omics Data Encyclopedia (NODE) and NCBI GenBank Sequence Read Archive (SRA) under project ID OEP003205 (https://www.biosino.org/node/project/detail/OEP003205, accessed on 13 and 14 March 2022) and accession number PRJNA816657 (https://www.ncbi.nlm.nih.gov/sra/PRJNA816657, accessed on 19 March 2022), respectively.

## Supplementary Materials

The following supporting information can be downloaded at: https://www.mdpi.com/article/10.3390/life12070954/s1, Figure S1: Variations in physicochemical parameters clustered by different stages in the WWTPs. Dunn’s test was used to analyze the specific variations between the specific groups considered significantly different (*p* < 0.05) based on Kruskal–Wallis tests. Letters ‘a’ and ‘b’ indicate significant differences (*p* < 0.05); Figure S2: Relative abundance and distribution of nitrifiers in samples from the aeration stage of WWTPs. (**a**) Relative abundance and structure of the top ten genera. (**b**) Composition and distribution of *Nitrospira* and *Nitrosomonas*. The relative abundance bars represent the ratio of the community to all selected bacterial communities instead of all reads; Figure S3: Spearman correlations analysis between environmental factors and the relative abundance of nitrifiers. “*” indicates the correlation was significant (*p* < 0.05); Table S1: Primer sets used for qPCR in this study [72,73,74]; Table S2: Primer sets used for high-throughput sequencing in this study [75,76]; Table S3: Number of comammox sequences at the aeration stage based on partial nested PCR results.
