# Supplementary material for: Abundance and Niche Differentiation of Comammox in the Sludges of Wastewater Treatment Plants That Use the Anaerobic–Anoxic–Aerobic Process
Source: Life (Basel). 2022 Jun 24;12(7):954. doi: 10.3390/life12070954 (PMC9322089; doi:10.3390/life12070954)
Supplement: Supplementary file 1 [file life-12-00954-s001.zip › life-1756406-supplementary.pdf]

Abundance and Niche Differentiation of Comammox in the Sludges of Wastewater Treatment Plants That Use the Anaerobic–Anoxic–Aerobic Process

Sheng-Nan Zhang, Jian-Gong Wang, Dan-Qi Wang, Qiu-Yue Jiang * and Zhe-Xue Quan *

Ministry of Education Key Laboratory for Biodiversity Science and Ecological Engineering, National Observations and Research Station for Wetland Ecosystems of the Yangtze Estuary, Institute of Biodiversity Science and Institute of Eco-Chongming, School of Life Sciences, Fudan University, Shanghai 200433, China; 19210700131@fudan.edu.cn (S.-N.Z.); 14110700066@fudan.edu.cn (J.-G.W.); 18110700020@fudan.edu.cn (D.-Q.W.)

* Correspondence: 15110700011@fudan.edu.cn (Q.-Y.J.); quanzx@fudan.edu.cn (Z.-X.Q.); Tel.: +86-21-3124-0665 (Z.-X.Q.)

Table S1. Primer sets used for qPCR in this study.

Target gene	Primers	Sequence (5' - 3')	Annealing temperature (°C)	Reference
16S rRNA gene	338F	ACTCCTACGGGAGGCAGC	55	[72, 73]
	536R	GTATTACCGCGGCKGCTG[1]		
AOB *amoA* gene	amoA1F	GGGGTTTCTACTGGTGGT	57	[56]
	amoA2R	CCCCTCKGSAAAGCCTTCTTC		
AOA *amoA* gene	Arch-amoAF	STAATGGTCTGGCTTAGACG	53	[74]
	Arch-amoAR	GCGGCCATCCATCTGTATGT		
comammox clade A *amoA* gene	C378AF	GTGGTGGTGGTCBAAYTA	55	[37]
	C576R	GAAGCCCATRTARTCNGCC		
comammox clade B *amoA* gene	C378BF	GTACTGGTGGGCBAAYTT	55	[37]
	C576R	GAAGCCCATRTARTCNGCC		
Nitrospira cynS gene	Ntspa-cynSF	TSATCGGHGTSTAYGGMGA	55	[37]
	Ntspa-cynSR	CCGTTCARSGTRATCTTGCA		

[1] Degenerate bases: K, G/T; S, G/C; B, G/C/T; Y, C/T; R, A/G; N, A/T/C/G; H, C/A/T; M, A/C.

Table S2. Primer sets used for high-throughput sequencing in this study.

Target	Primers	Sequence (5' - 3')	Annealing temperature (°C)	Reference
V1-V2 regions of bacterial 16S rRNA gene	27FYM	XXXXXXXXXXXX-TCAGAGTTTGATYMTGGCTCAG[1 2]	55	[75]
	338R	TGCTGCCTCCCGTAGGAGT		[76]
	338R-PV[3]	TGCWGCCACCCGTAGGTGT		This study
partial nested PCR	A189Y	GGNGACTGGGAYTTYTGG	55	[12]
	CA209	GAYTGGAARGAYCGNCA		
	C576R	XXXXXXXXXXXX-GAAGCCCATRTARTCNGCC		

[1] Degenerate bases: Y, C/T; M, A/C; W, A/T; N, A/T/C/G; R, A/G.
[2] XXXXXXXXXXXX represents the 12-bp barcodes used to differentiate the samples in the high-throughput sequencing library mixture.
[3] 338-PV and 338R were mixed at equal concentrations as reverse primers. The primer 338-PV was modified from primer 338R to improve the coverage of Planctomycetota and Verrucomicrobia in the samples.

Table S3. Number of comammox sequences at the aeration stage based on partial nested PCR results.

	clade A.1	clade A.2	clade B
A-O1	19,157	94	0
A-O2	17,581	36	0
B-O1	19,681	15	0
B-O2	14,809	69	14
C-O1	28,133	126	8
C-O2	13,741	201	0
D-O1	33,414	31	2
D-O2	45,535	21	5
E-O1	35,245	20	16
E-O2	42,623	40	0

Sum	269,919	653	45

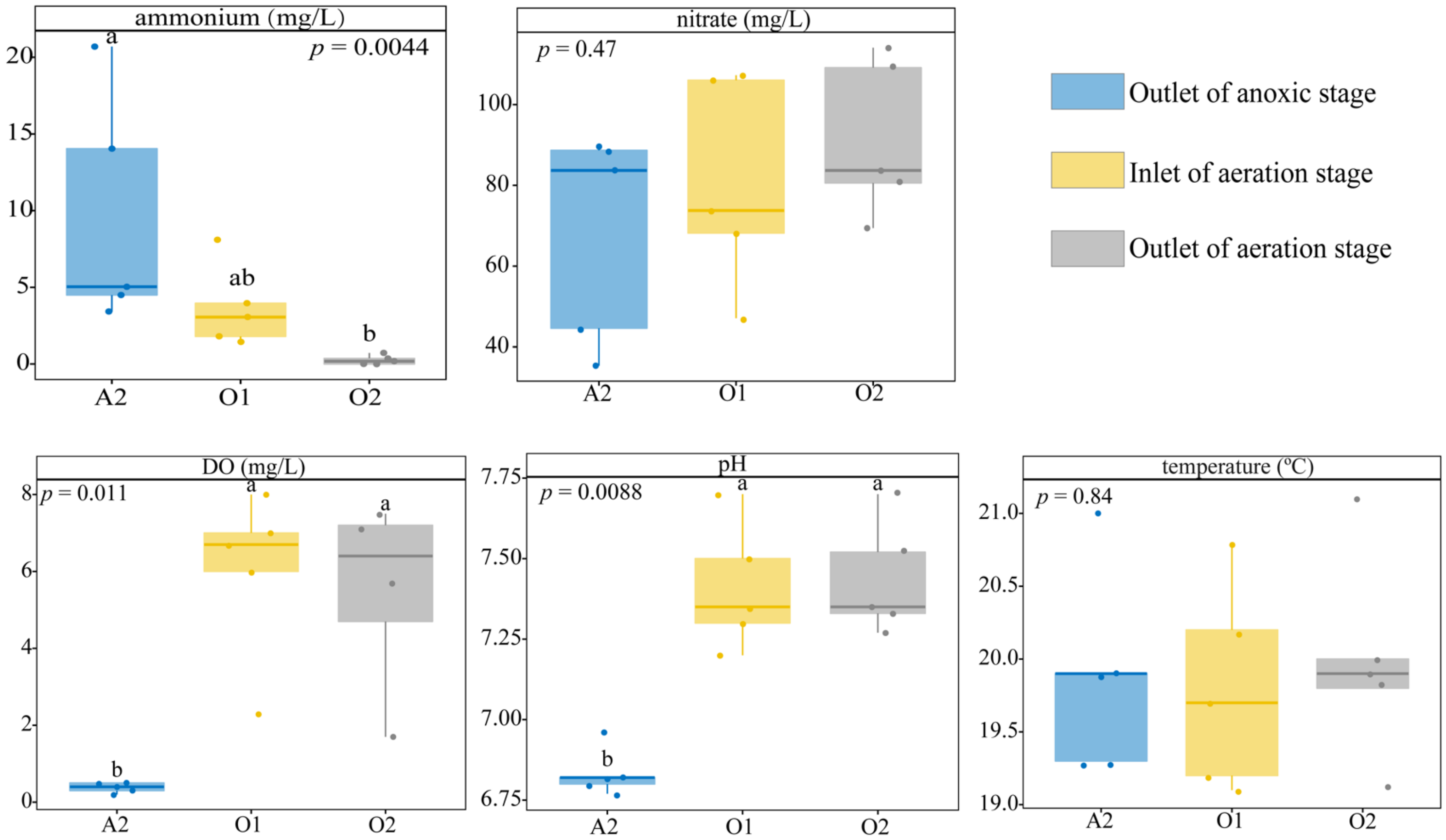

Figure S1. Variations in physicochemical parameters clustered by different stages in the WWTPs. Dunn's test was used to analyze the specific variations between the specific groups considered significantly different ($p < 0.05$) based on Kruskal–Wallis tests. Letters 'a' and 'b' indicate significant differences ($p < 0.05$).

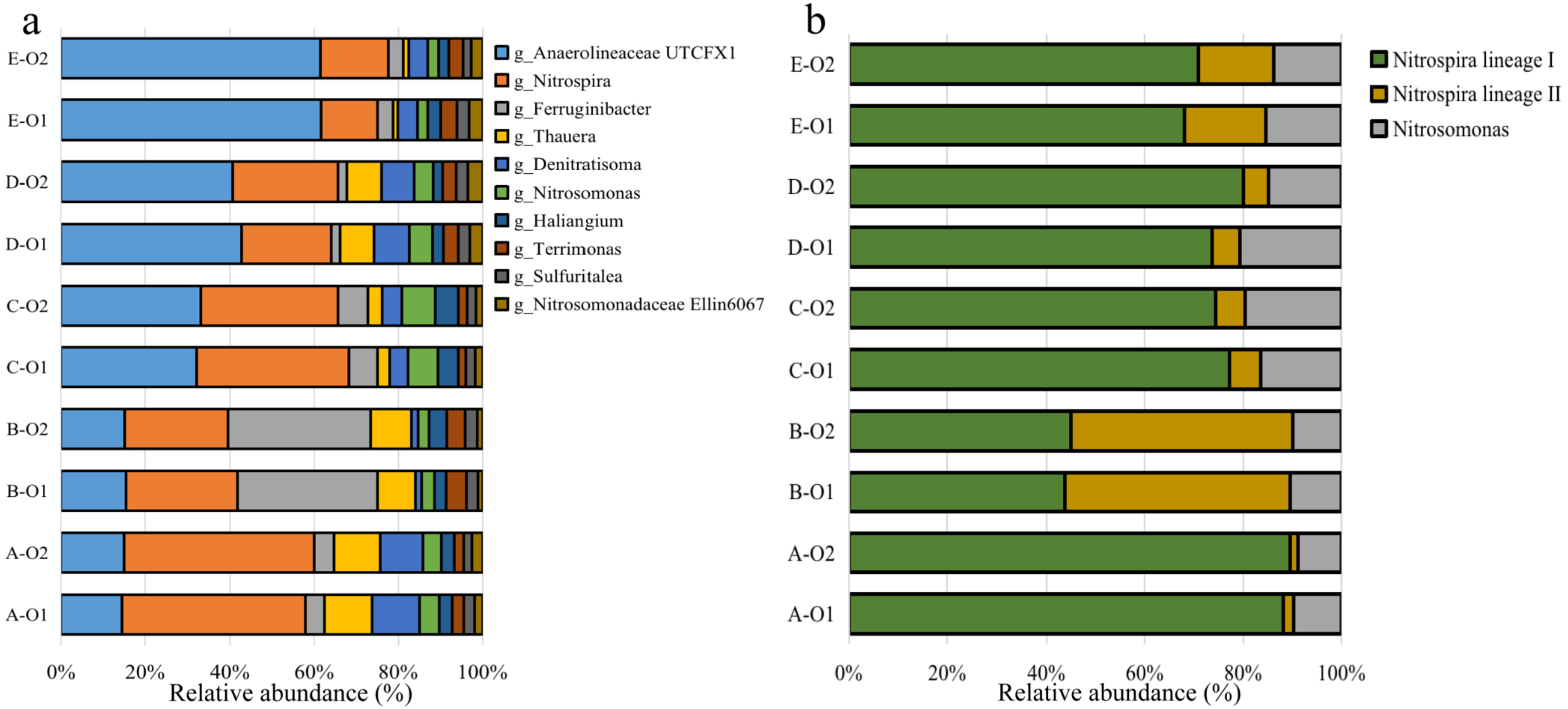

Figure S2. Relative abundance and distribution of nitrifiers in samples from the aeration stage of the WWTPs. **(a)** Relative abundance and structure of the top ten genera. **(b)** Composition and distribution of *Nitrospira* and *Nitrosomonas*. The relative abundance bars represent the ratio of the community to all selected bacterial communities instead of all reads.

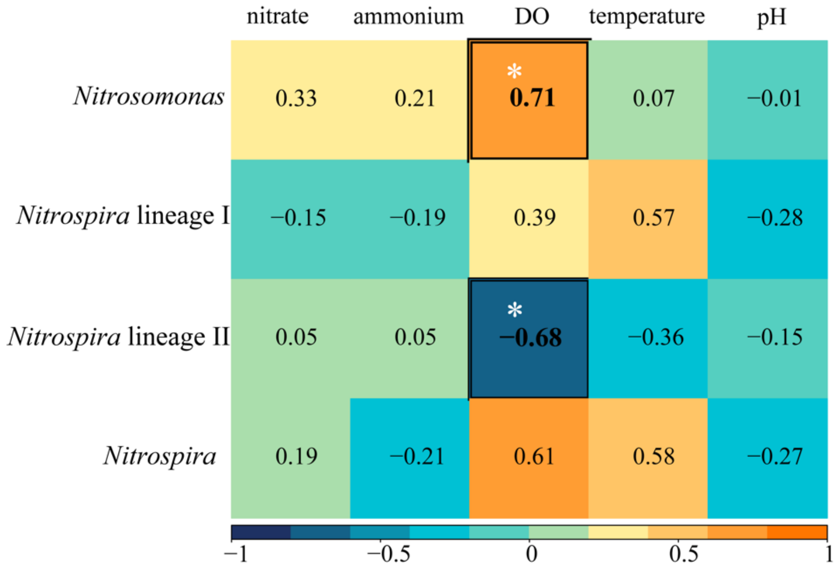

Figure S3. Spearman correlations analysis between environmental factors and the relative abundance of nitrifiers. "*" indicates the correlation was significant ($p < 0.05$).
